# Frozen Mummies from Andean Mountaintop Shrines: Bioarchaeology and Ethnohistory of Inca Human Sacrifice

**DOI:** 10.1155/2015/439428

**Published:** 2015-08-06

**Authors:** Maria Constanza Ceruti

**Affiliations:** Instituto de Investigaciones de Alta Montaña, Universidad Católica de Salta, Campus Castañares, 4400 Salta, Argentina

## Abstract

This study will focus on frozen mummies of sacrificial victims from mounts Llullaillaco (6739 m), Quehuar (6130 m), El Toro (6160 m), and the Aconcagua massif. These finds provide bioarchaeological data from mountaintop sites that has been recovered in scientifically controlled excavations in the northwest of Argentina, which was once part of the southern province of the Inca Empire. Numerous interdisciplinary studies have been conducted on the Llullaillaco mummies, including radiological evaluations by conventional X-rays and CT scans, which provided information about condition and pathology of the bones and internal organ, as well as dental studies oriented to the estimation of the ages of the three children at the time of death. Ancient DNA studies and hair analysis were also performed in cooperation with the George Mason University, the University of Bradford, and the Laboratory of Biological Anthropology at the University of Copenhagen. Ethnohistorical sources reveal interesting aspects related to the commemorative, expiatory, propitiatory, and dedicatory aspects of human sacrifice performed under Inca rule. The selection of the victims along with the procedures followed during the performance of the *capacocha* ceremony will be discussed, based on the bioarchaeological evidences from frozen mummies and the accounts recorded by the Spanish chroniclers.

## 1. Introduction

The practice of human sacrifice has been known to occur cross-culturally throughout history. Humans have been sacrificed in order to celebrate special events, to mark royal funerals, in response to natural disasters, to atone for sins committed, to consecrate a special construction project or location, and to ensure fertility and health ([1]: 290; [2]). Sacrificial victims have also been executed in oer to serve as retainers to high-ranking individuals in the afterlife. Inca human offerings should therefore be considered as being commemorative, expiatory, propitiatory, and or dedicatory sacrifices.

During the Late Post Classic Period in ancient Mesoamerica, the Aztecs embarked upon the practice of ritual of human sacrifice involving the removal of hearts in epic proportions (rooted in the Maya-Toltec tradition) based on their belief that human blood needed to be continuously offered to the Sun deity lest the god grow weak and not be able to continue his journey through the sky each day. Sacrifices celebrating the completion of special constructions were conducted for such projects such as the dedication of the twin temples of Tlaloc and Huitzilopochtli in Mexico-Tenochtitlan. The Maya-Toltec sacrificial rites were not limited to the removal of the heart; they also included the flaying of the victim as well as the eating of his flesh ([3]: 51). The cruelty of these very public sacrificial events presumably served to reinforce the power of the Aztecs in the minds of allies as well as potential rivals.

In the ancient South American Andes, evidence of human sacrificial practices has been found depicted on Moche pottery (100 to 700 AD). The iconography of human sacrifice taking place on mountains seems to have been related to agricultural fertility rites and to the management of water resources ([4]: 35-36). But it was almost eight centuries later, under the rule of the Inca civilization, that the practice of human sacrifice on mountaintop shrines reached its highest level of cultural elaboration and expression.

The Inca Empire spread from its capital at Cuzco (located heartland in the Peruvian highlands) northwards to southern Colombia and as far south as central Chile. Since its beginning in 1438 A.D. until the Spanish conquest in 1532 A.D. (a span of less than one hundred years), the* Tawantinsuyu* achieved the highest level of sociopolitical organization in the history of Andean civilization. During this period, the Incas constructed shrines on the summits of snow-capped peaks (over 5,000 m in elevation). These remote locations became the settings for the ritual performance of human sacrifice. The extraordinary preservation of the victims' bodies as well as of many of the grave goods recovered in the extremely cold and dry high elevation Andean environment provides exceptional bioanthropological evidence (both osteological as well as soft tissue) for the study of human sacrifice among the Incas.

The frozen bodies of three Inca children were discovered by Johan Reinhard and the author of this paper on the summit of mount Llullaillaco, at an elevation of 6715 m, at the world's highest archaeological site. They are considered to be among the best preserved Precolumbian mummies known to date. They have been previously analyzed in the context of the diversity of mummies worldwide [5]; as objects of dedication [6], in connection to the religious role of children in the Andes [7], as actors in the ceremonies of* capacocha* [8, 9], and as objects of paleopathological and medical research [10].

Numerous interdisciplinary studies were conducted on the frozen mummies from mount Llullaillaco during the years in which they were preserved at the Catholic University of Salta (see Figures 14 and 15). These studies included radiological evaluations by conventional X-rays and CT scans, which provided information about condition and pathology of the bones and internal organs; as well as dental studies oriented to the estimation of the ages of the three children at the time of death [11–13]. Ancient DNA studies and hair analysis were also performed in cooperation with academic institutions including the George Mason University, the University of Bradford and the Laboratory of Biological Anthropology at the University of Copenhagen [14–16].

The archaeology of mount Llullaillaco, including its architectural and landscape features, has been studied and published in the context of Inca religion and sacred landscape in the Andes [17–19]. The material offerings associated to the Llullaillaco mountaintop burials have been described and studied in terms of their social use and symbolic meaning [17]. Expert work has been undertaken on the technological analysis of the textile objects; and pottery items have been subjected to neuthron activation analysis to help identify their geographical origin. The results of pottery analysis have demonstrated that the ceremonial items buried together with the Llullaillaco mummies had been manufactured in Cusco and around Lake Titicaca, while some of the pottery found at the foot of the volcano came from northern Chile [20]. This is in with what would be expected in the context of a state sponsored pilgrimage of* capacocha,* in which ceramic offerings carried to the summit of a mountain were customarily brought from Cusco or the Bolivian highlands, while utilitarian pottery used at base-camp could be of local origin.

This study will focus on the frozen mummies of sacrificial victims from mount Llullaillaco (6739 m), volcano Quehuar (6130 m), mount El Toro (6160 m), and the Pyramid of the Aconcagua massif. These finds provide a robust and valuable amount of bioarchaeological data from Inca mountaintop shrines that has been recovered in scientifically controlled excavations in the northern and western regions of Argentina. Reference will also be made to mummies excavated under nonscientifically controlled digs from Mt. Chañi and Mt. Chuscha in Argentina, and from Mt. El Plomo and Cerro Esmeralda in Chile. These peaks are mainly located in areas of modern day northwestern Argentina, as well as north and central Chile, and were once part of the southern province of the Inca Empire, known as* Collasuyu*.

Ethnohistorical sources reveal interesting aspects related to the commemorative, expiatory, propitiatory, and/or dedicatory aspects of human sacrifice performed under Inca rule. The selection of the human victims along with a description of the procedures followed during the performance of the sacred* capacocha* ceremony will also be discussed, based on the bioarchaeological evidences from frozen mummies and the accounts recorded by the Spanish chroniclers.

## 2. Inca Human Sacrifice in the Historical Sources

An Inca* capacocha* ceremony involved the movement of sacrificial victims (along with various ceremonial goods) from the peripheral communities of the conquered provinces towards the centrally located capital city of Cuzco. After being ritually transformed into Inca-style offerings in the capital city, the sons and daughters of local chiefs, as well as the chosen Virgins of the Sun god, were then made to travel in ritual procession, to various sacred places located throughout the empire. They eventually would be sacrificed and buried at designated locations as vivid representations of the state cult to the sun god Inti, as well as an imperial homage to the local sacred sites, known as* huacas*.

The term* capacocha* consists of two Quechua words: “*capac*” meaning “royal” and “*cocha*” the word for “lake” or “body of water” ([21]: 134, 559). According to Reinhard [22], the* capacocha* would serve as a powerful public manifestation of the Inca's power to organize such propitiatory rituals. Based on the fact that the Quechua word* hucha* can signifies “sin,” interpreted “*capac-hucha*” as the ritual procedure designed to atone for transgressions committed and to prevent misfortune that could affect the Inca ruler and his empire.

Human sacrifice being strictly under the control of the Inca State, ethnohistorical sources specify that the ritual killing of native Andean people could only occur with the explicit approval of the emperor:

*…Cuando el dicho Inca quería hacer algún sacrificio y aplacar alguna guaca […] entonces por su orden mataban algunos indios y los sacrificaban a los cerros y guacas que enviaba a mandar el dicho Inca, y que sin su orden no podían sacrificar indios ([23]: 330). *

### 2.1. Celebratory Sacrifices

Certain events of the life of the emperor were celebrated by means of human sacrifices. The chroniclers report that sacrifices took place upon the coronation of a new Inca ruler ([24]: 92; [25]: 26). Reportedly, two hundred children were sacrificed when a new ruler assumed the throne ([26]: 87) and even the birth of the son of the emperor was celebrated with sacrifices ([27]: 93). Other important events, such as the victorious return of the Inca from battle, called for the sacrifice of war prisoners in thanksgiving to the Sun God Inti for a successful military campaign ([28]: 143).

### 2.2. Funerary Sacrifices

Under the Inca rule, ritual killing of wives and servants was performed as* necropompa*, in order to provide a deceased emperor with a fitting cadre of servants in the afterlife. Chroniclers report that 1,000 persons were sacrificed upon the death of Inca Emperor Huayna Cápac ([29]: 118), although other sources mention that the number of victims was over 4,000 ([30]: 80). According to chronicler Bernabe Cobo, many victims would voluntarily offer to be sacrificed so that they could join those servants who had already been killed in order to serve the deceased ruler in the hereafter ([28]: 161). Chronicler Santa Cruz Pachacuti refers to the ritual homicide of wives and servants as an example of commemorative sacrifices undertaken by the Inca State after the death of an emperor:

*Se entierra a muchos yanas, mujeres y criados amados del dicho Inca; todos estos eran escogidos, entendiendo que el Inca había de ser servido en la otra vida* ([31]: 306).

### 2.3. Sacrifices of Atonement in Response to Natural Disasters

Sacrifices were often conducted in response to natural calamities, such as droughts, epidemics, earthquakes, and volcanic eruptions ([32]: 112; [33]: 155; [34]: 48). According to a Spanish chronicler:

…*el sacrificar niños u hombres era para cosa de gran importancia, como pestilencia grande o mortandad*… ([29]: 193).

These acts of atonement involving human sacrifice were based on their belief that illness and natural disasters were forms of supernatural punishment for sins committed or for the improper performance of various sacred rituals. Only a major offering such as a human life could serve to reestablish the spiritual equilibrium with the cosmos ([35, 36]: 426-427):

*Tenían por opinión que todas las enfermedades venían por pecados que se hubiesen hecho. Y para el remedio usaban de sacrificios* ([29]: 12).

Chronicler Martín de Murúa ([34]: 48) reports that during the devastating eruption of the Misti Volcano in 1440, Mama Ana Huarque Coya, queen and wife of Emperor Pachacutec Inca Yupanqui, ordered many sacrifices to be performed in various temples throughout Cuzco in the hopes that this would assuage the supernatural lord's (*apu*) anger. The volcanic activity ceased only after Pachacutec himself traveled with several priests and shamans to the massif (located near Arequipa, Peru) to personally make sacrificial offerings designed to calm the divine fury of the mountain:

*Así mostró su incomparable ánimo y ser en un terrible terremoto que hubo en su tiempo en la ciudad de Arequipa, resultado de un volcán temeroso que está a tres leguas de la dicha ciudad. El cual cuentan los indios que lanzó de si tanto fuego y con tan espantosas llamaradas, que en muchas leguas quedaron los indios atónitos y absortos. Fue cierto haber revocado y salido del volcán tanta ceniza que llovió en todo el reyno, con universal admiración y miedo. Si no fuera por el ánimo de esta Coya Mama Ana Huarque se hubiera asolado la mayor parte de la gente de todas las provincias cercanas a Arequipa. La cual mando lo primero hacer grandísimos sacrificios a sus ídolos en el templo que ellos llaman Tipci Huaci, que quiere decir casa del universo, y en otros muchos que había en el Cuzco, hasta que sabido por el Inga Yupanqui, su marido, vino con suma prisa dentro de pocos días y se partió a Arequipa con muchos pontífices, adivinos y hechiceros, y llegado cerca, hizo diversos sacrificios*… ([34]: 48).

### 2.4. Sacrifices to Consecrate a Special Construction Project or Location

Chronicler Betanzos reports that the construction of the Temple of the Sun in Cuzco was preceded by* capacocha* ceremonies in which several children sent by the local rulers were buried alive in the sanctuary as a way to consecrate the “sacred space” of the place of worship ([27]: 46). According to the chronicler Rodrigo Hernández Príncipe [37], the sacrifice and burial of a ten-year-old girl on a summit near her natal village was performed to assure successful completion of a new irrigation canal. Some human sacrifices may also have served as a means of establishing boundaries along the periphery of the ever expanding Inca Empire ([38]: 551; [39]: 371; [40]: 18).

### 2.5. Fertility and Health Sacrifices

The Incas dedicated human offerings to the Sun god Inti, to the Weather (Thunder) deity Illapa, and to the Creator god Viracocha, as well as to local deities (*huacas*) in order to ensure the fertility of the crops and to plead for favorable weather as well as to petition for the good health of the emperor. In the words of Cobo:

*They made sacrifices to the Sun so that he would make the plants grow, to the Thunder, so that he would make it rain and not hail or freeze, and to the rest of the special gods and second causes. First they would speak with Viracocha and afterwards they would speak with the special gods. And in their sacrifices to all the universal huacas they would plead for the health of the Inca* ([32]: 111).

In 1571, a group of Inca men informed the Spaniards that the* capacocha* offerings were made in order to ensure favorable weather for crops ([33]: 155). Reinhard and the author have extensively analyzed the symbolic link between the worship of sacred mountains, by means of offerings and sacrifices, and the propitiation of fertility in ancient and modern times in the Andes [19, 41].

## 3. Bioarchaeological Evidence of Inca Human Sacrifice on High Andean Mountains

The climax of the Inca* capacocha* ceremony involved the sacrifice of children and their burial with lavish grave goods, such as* cumbi* textiles, fine pottery, and figurines made of gold, silver and* Spondylus* shell. After the long processions to the sacred summits, these children would be sacrificed and thus be sent on a supernatural pilgrimage in the hereafter where they served as messengers to the world of the ancestors and deities.

Archeologically, the sacrifices performed in the context of an Inca* capacocha* can be identified by (a) the location of the burial site (often on mountaintops), (b) the age and sex of the victims, usually boys and girls under the age of ten, and/or (c) teenage women who were part of the so called “*acllas*” or “chosen ones.” Inca offerings for* capacocha* ceremonies include pottery of imperial style, textiles, and miniature figurines made of metal and shell, representing individuals of the same sex as the victim sacrificed (Figure 16). Alternatively, other kind of human sacrifices, such as those performed during the* necropompa* of a dead emperor, might include victims of older ages, selected among the spouses, concubines, and servants of the Inca.

**Figure 1:**
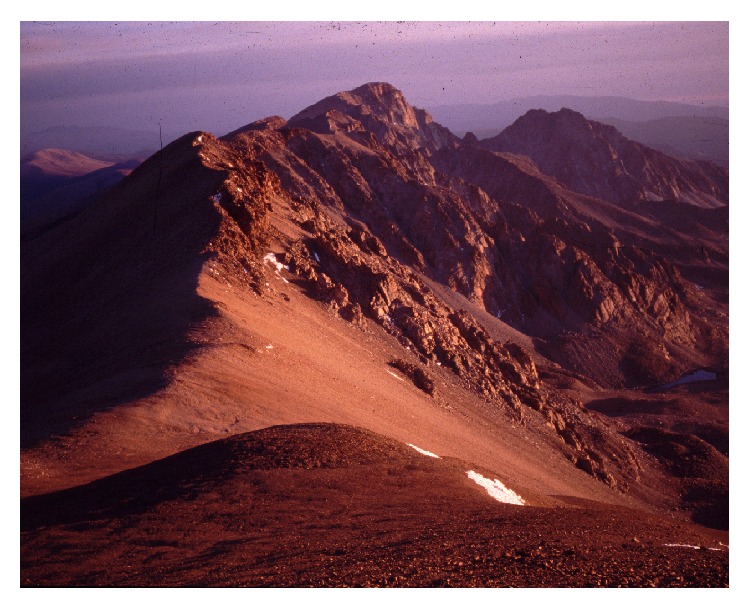
Mount Chañi in northern Argentina (© Constanza Ceruti).

One of the earliest sources of evidence for Inca human sacrifice on mountaintop shrines came from a case of looting that occurred in 1905, when the naturally mummified body of a child was extracted from the summit of Mt. Chañi, a 5896 m peak located in northern Argentina (Figure 1). Items included with the mummy were tunics, two belts, one comb, one bag covered in feathers and a pair of sandals [42, 43]. Various archaeological surveys were conducted by the author on the Chañi massif during 1996 and 1997 [44], and three years later, research at this same site was directed by Johan Reinhard and the author. This investigation resulted in the discovery of the burial place of the Chañi infant [45].

**Figure 2:**
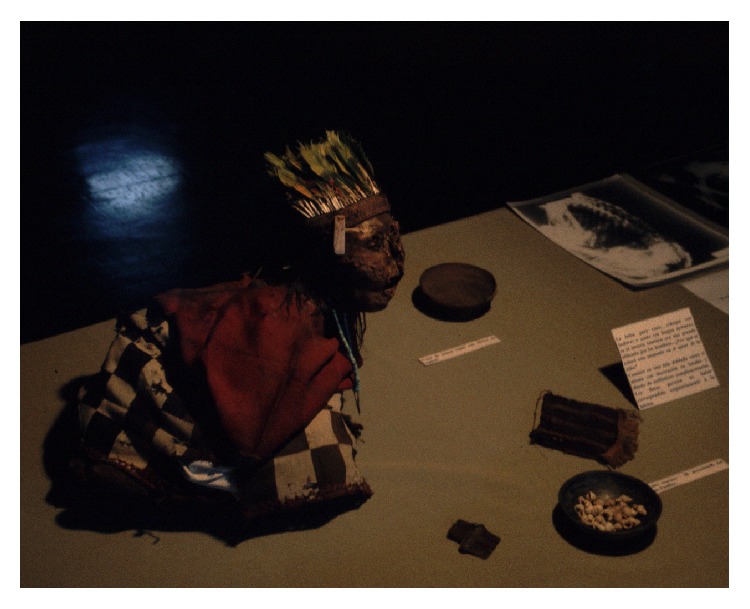
Mummy from mount Chuscha (© Constanza Ceruti).

In 1922, looters removed the body of a young female mummy that was buried with associated offerings including a* cumbi* tunic, a textile bag, one belt, three combs, and a feather adornment made from tropical birds on her head [46]. Interdisciplinary research on the Chuscha mummy (Figure 2) was later coordinated by Dr. Juan Schobinger, Emeritus Professor of Archaeology at the National University of Cuyo [47]. The mummy had been extracted from one of the summits of Mt. Chuscha (over 5300 m), in northwestern Argentina. The burial site was relocated during a high-altitude survey by the author in 1996 [48].

It was the discovery by treasure hunters in 1954 of the frozen body of an 8-year-old boy on Mt. El Plomo in central Chile that captured the attention of the archaeological community. Although the artifacts were not recovered in a scientifically controlled excavation, they did yield a substantial amount of valuable data [49]. The child seems to have died of exposure to the cold or may have even been buried alive [50].

**Figure 3:**
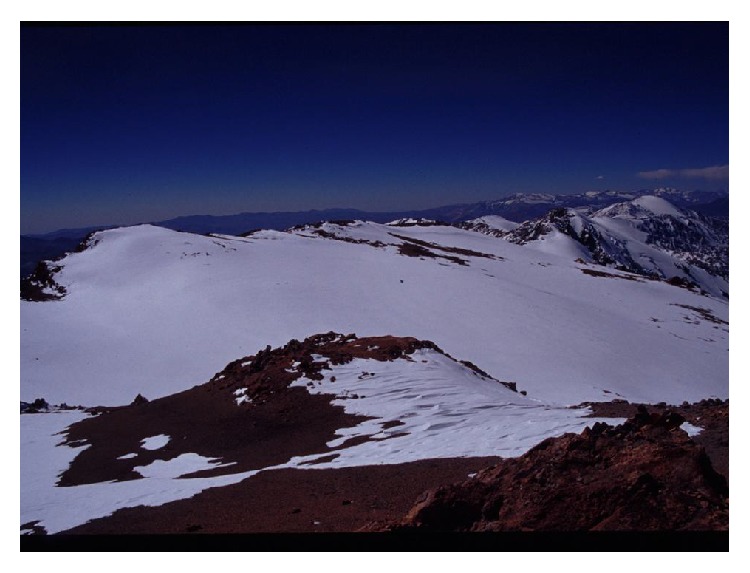
Summit of mount El Toro (© Constanza Ceruti).

**Figure 4:**
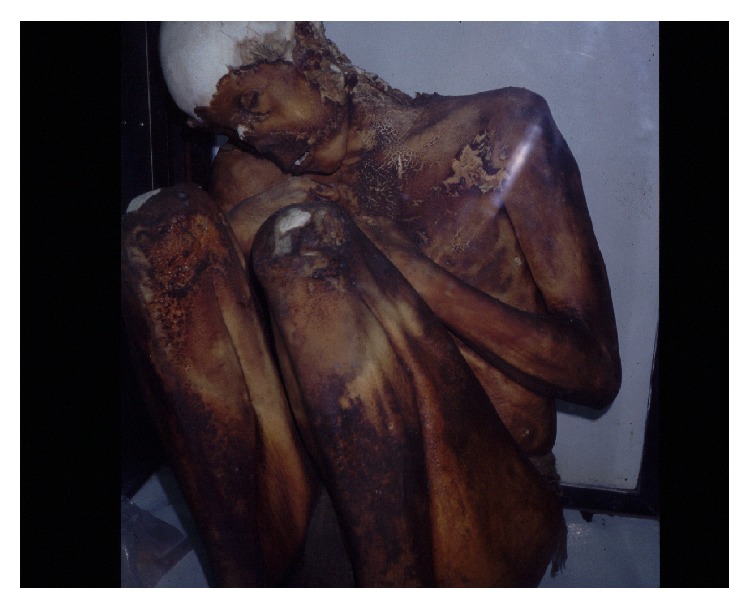
Mummy from mount El Toro (© Constanza Ceruti).

In 1964, mountain climbers Antonio Beorchia Nigris and Erico Groch came across the frozen body of an adult male that was found inside a circular stone structure (located near an Inca ceremonial rectangular structure) at over 6000 m in elevation on Mt. El Toro (Figure 4) in western Argentina. Archeologist Juan Schobinger recovered the mummy and conducted an investigation which revealed that the man had apparently been killed by strangulation. Wearing only a breechcloth, the victim had been buried with a set of objects including a mantle, two ordinary tunics, a woolen cap, two pair of sandals, a sling, and feathers [35]. A more recent survey of the southern slopes and summit of El Toro (Figure 3) did not reveal further traces of the prehispanic use of ritual space on the mountain [51].

In 1976, a road construction crew came across the burial of two females (1 child and 1 adult) near the summit of Mt. Esmeralda found along the north coast of Chile. Although this mountain is relatively low in elevation, it nevertheless still dominates the coast when viewed from the ocean. The human remains found at the site had been preserved by desiccation and many artifacts were found in association with the mummies, albeit not in a scientifically controlled excavation. Nonetheless, the items recovered, which included* cumbi* cloth, fine Inca pottery, and* Spondylus* shell, clearly indicated that this burial was a* capacocha* offering and furthermore, the victims seemed to have been killed by strangulation [52, 53].

**Figure 5:**
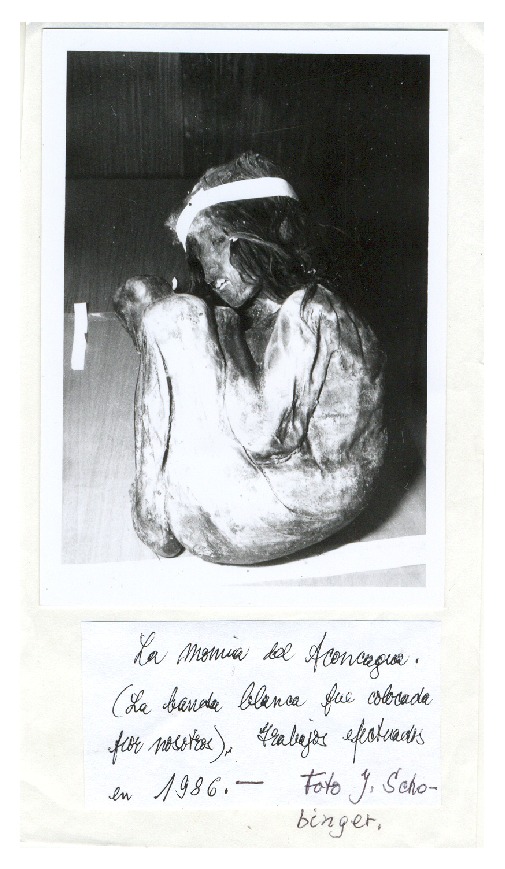
Mummy from mount Aconcagua (© Constanza Ceruti).

In 1985, mountain climbers came across the frozen body of a seven-year-old boy who was found inside a semicircular stone structure (over 5300 m in elevation) on the slopes of Mt. Piramide located in the Aconcagua massif of western Argentina (Figure 5). The mummy and the offerings associated with it were recovered and studied by a team of scholars led by Juan Schobinger. The victim had been killed by a blow to the head, although he also had ribs broken by compression during the process of bundling the body. He was dressed in two tunics, wearing sandals and a necklace of stones. The bundle contained several textile mantles (one was covered in feathers of highland birds) and a feathered headdress, woven belts, and five tunics, some of them woven in* cumbi*, three breechcloths, a pair of sandals, and two bags. Inside the tomb were found three male figurines made of gold, silver, and* Spondylus* shell, which were adorned with feathers of rainforest birds. In addition to these items, two llama figurines made of* Spondylus* shell and a silver figurine representing a camelid were found [54]. Red pigment was identified on the boy's skin, and on his tunic, traces of which were found in the victim's vomit and feces [55].

**Figure 6:**
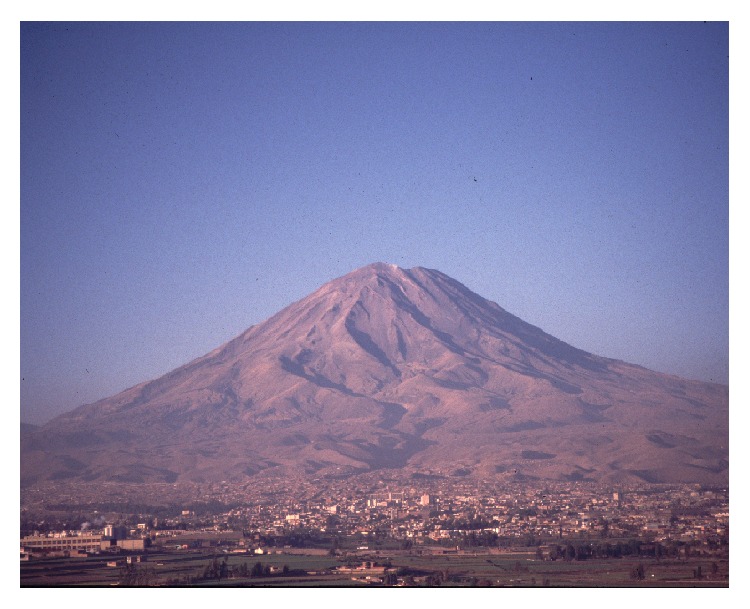
Volcano Misti in Arequipa (© Constanza Ceruti).

Mt. Misti is a steep sided, cone shaped volcano (5,822 m in elevation) found near the city of Arequipa in southern Peru (Figure 6). It is located in what once was the western Inca province of* Cuntisuyu*, (the Ampato and Sara Sara Volcanoes are also found in this region). Misti is one of the few active volcanoes that erupted during the historic Inca period, and various chroniclers (mentioned above) report that both ritual offerings and sacrifices were conducted at this site in response to this activity. In 1998, Johan Reinhard, José Chavez, Constanza Ceruti and a team of collaborators, excavated the Inca ceremonial site located inside the volcano's crater, which is marked by unimpressive rectangular and circular alignments of stones. The recovery of the bodies of six human sacrificial victims and more than forty statuettes (one of the largest* capacocha* assemblages ever found) demonstrated the importance of this site to the Incas [19]. Unfortunately, the preservation of the bodies and textile offerings on Misti was very poor, due to the high concentration of sulfur in the soil and the high temperatures within the crater.

**Figure 7:**
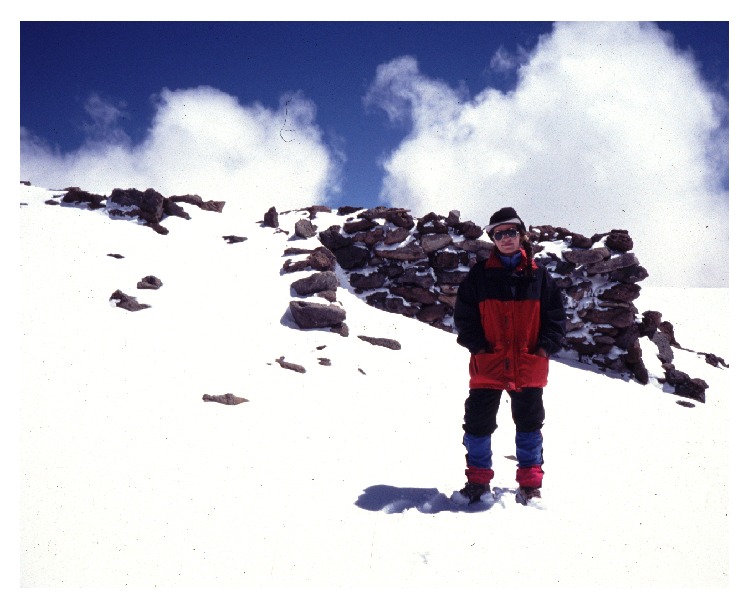
Inca platform on the summit of mount Quehuar (© Constanza Ceruti).

Mt. Quehuar, a 6130 m extinct volcano in the highlands of northwestern Argentina, holds one of the most impressive Inca mountaintop shrines in the Andes (Figure 7). The summit's architectural complex included an artificial raised platform (*ushnu*) measuring over 6 m long and 1.7 m high, with a frontal ramp, as well as a circular stone structure with walls more than 2.2 m high and 1.2 m thick [19]. In 1999, Johan Reinhard, José Antonio Chávez and the author directed a National Geographic Society sponsored excavation of this particular Inca shrine. A human sacrificial victim had been originally buried inside the circular structure, but looters had employed dynamite to gain access to the tomb. The blast left scattered pieces of the offerings including textiles, fragmented pottery, corn seeds, meat, and bones from a sacrificed camelid strewn about the site. A typical Inca style female figurine made of* Spondylus* shell and dressed in miniatures of* cumbi* clothes was recovered from the* ushnu* platform [56]. Apparently, a small damaged tunic had also been found at the same structure in 1974 ([57]: 188–200). Subsequent DNA analysis conducted on the individual buried on the summit of Mt. Quehuar revealed that it was female [58] but given the poor preservation of the remains (stemming from the use of explosives in the recovery of the body), it was impossible to establish the cause of death. However, due to the types of artifacts recovered in association with the body, it is parsimonious to conclude that this was a* capacocha* sacrifice. The victim was about twelve years old at the time of her sacrifice, according to estimations based on the length of her long bones, as they appeared in the X-rays.

**Figure 8:**
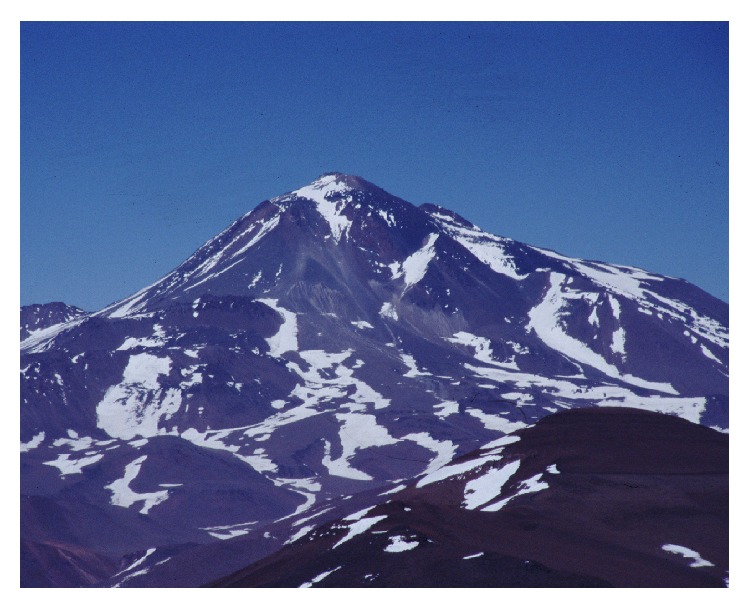
Volcano Llullaillaco in northern Argentina (© Constanza Ceruti).

**Figure 9:**
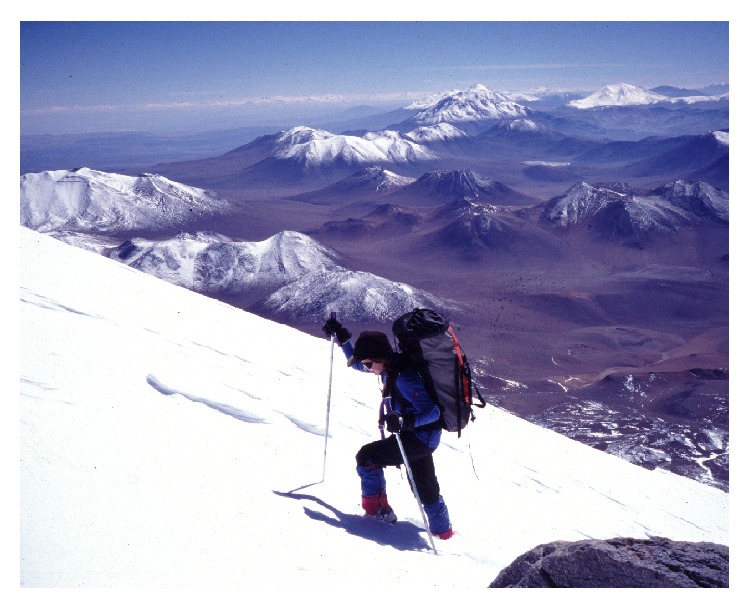
The author climbs to the top of volcano Llullaillaco (© Constanza Ceruti).

**Figure 10:**
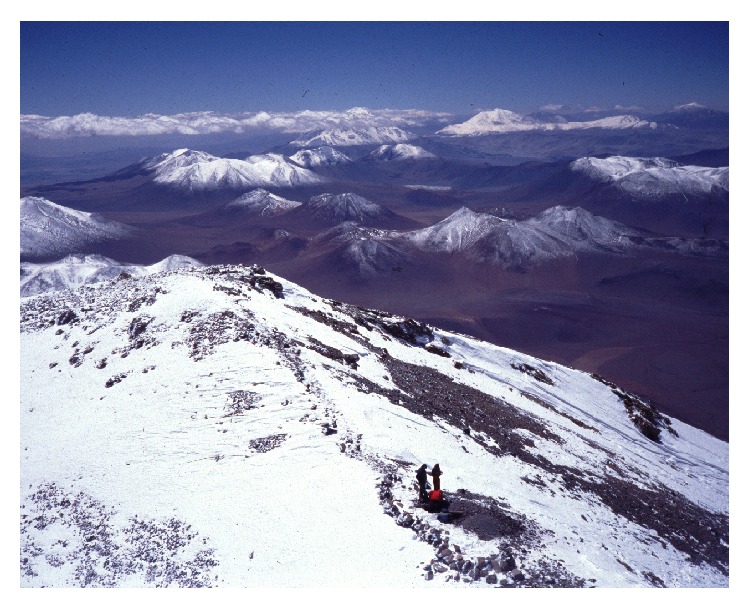
Inca shrine on the summit of mount Llullaillaco, the highest ceremonial site in the world (© Constanza Ceruti).

On the summit of Mt. Llullaillaco (6739 m), in northwestern Argentina (Figure 8), Johan Reinhard and the author of this paper directed an archaeological expedition funded by the National Geographic Society, which took place in March 1999 (Figure 9). At an elevation of 6.715 m, the summit shrine of Llullaillaco is the world's highest archaeological site (Figure 10). Excavations at the main ceremonial structure (a rectangular stone platform) revealed three separate burials and several offering assemblages. One young woman, one girl, and one boy were found together with more than one hundred offerings comprising metal figurines,* Spondylus* shell, fine imperial pottery,* cumbi* textiles, and feathered adornments of tropical birds, all in an excellent state of preservation (cf. [19, 59]).

The Llullaillaco boy, who was 7 years old, was wearing a red tunic, leather moccasins, fur anklets, a silver bracelet, and a sling wrapped around his head, with his forehead adorned with white feathers (Figure 13). Two figurines were found, one representing a man and the other a llama, which had been placed on the ground, close to the body. An aryballo (an Inca ceramic vessel) that had contained* chicha* (an Andean corn beverage) was recovered in the fill of the tomb, as well as a* Spondylus* seashell [19]. The body of a six-year-old female had apparently been hit by lightning after she had been buried in the tomb. She was wearing a sleeveless dress and a shawl, both kept in place with metal pins, moccasins on her feet and a metal plaque on her forehead. Textile and ceramic items, as well as metal figurines, were placed around her body on the bottom of the tomb ([59]: 60-61).

The Llullaillaco maiden (Figure 12) was about 15 years old at the time of her sacrifice ([60]: 7). Her body was placed in the tomb facing northeast and was covered with two brown outer mantles. A feathered headdress was placed on her head and an exquisite* cumbi* tunic on her shoulder outside of the funerary bundle. Her hair was combed in numerous intricately woven braids. Her offering assemblage also included textile bags and belts, gold and silver figurines, and various ceramic items ([59]: 59-60). Excavations also brought to light several additional sets of offerings containing metal and* Spondylus* shell statuettes, some of which included two male figurines at the head of a row of metal and seashell figurines representing llamas.

## 4. Discussion 

Bioarchaeological findings of frozen mummies on the summits of high altitude Andean peaks indicate a high degree of correspondence with the ethnohistorical descriptions written by various chroniclers on the Inca ceremonies of human sacrifice with regards to the profile of the victims chosen and the sacrificial techniques involved. Ideological motifs that were invoked to legitimize the need for a* capacocha* ceremony would have contributed to the acceptance of the ritual violence exerted by the Inca Empire during its territorial expansion.

The chroniclers report that children were selected to serve as messengers to the gods because their “purity” made them the most fitting mediators between humans and deities ([61]: 233; [37]). The parents of sacrificial victims were expected to surrender their children willingly and were encouraged by the Inca to consider the selection of their offspring for* capacocha* as a great social honor as well as being an act of pious devotion ([32]: 112; [37]; [25]: 81).

Ethnohistorical sources state that the boys chosen for sacrifice during* capacocha* ceremonies were expected to be between four and ten years old ([62]: 342; [29]: 37; [28]: 235). Bioarchaeological findings are consistent with these reports as it has been determined that the Aconcagua and Llullaillaco boys were approximately seven years old at the time of their deaths.

**Figure 11:**
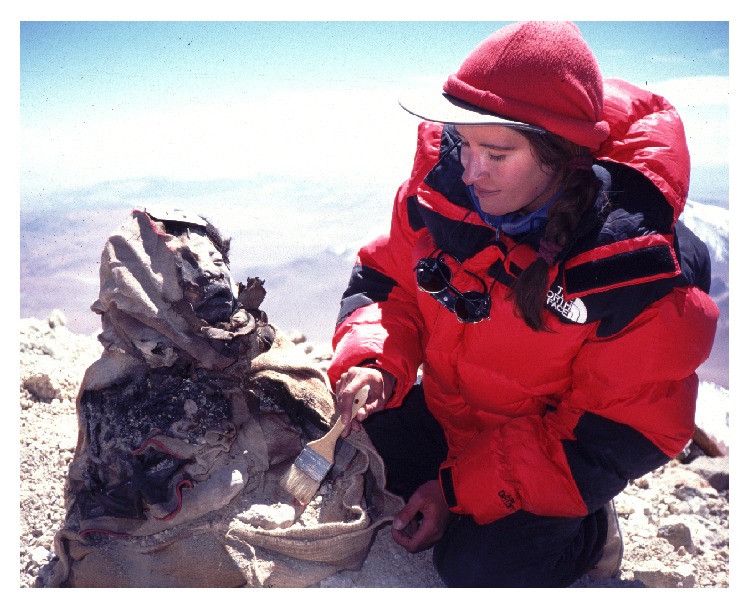
Constanza Ceruti discovering an Inca female mummy on the summit of Llullaillaco (© Constanza Ceruti).

**Figure 12:**
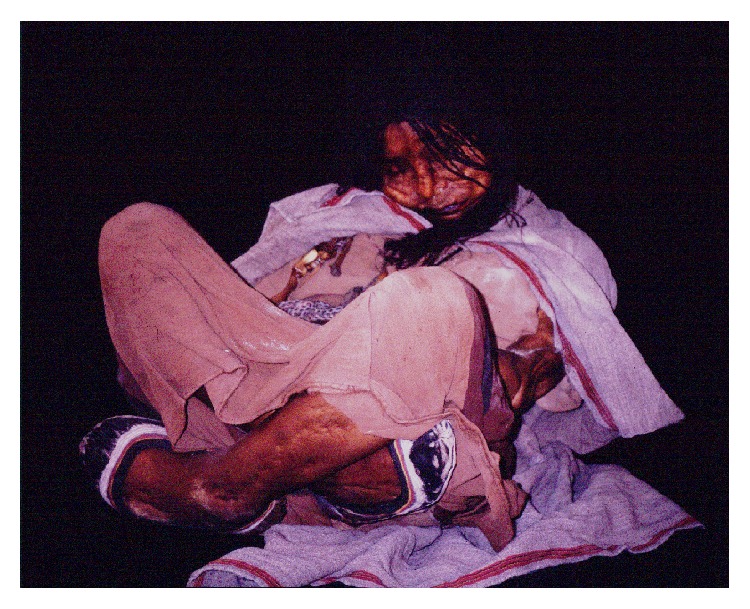
The Llullaillaco Ice Maiden (© Constanza Ceruti).

**Figure 13:**
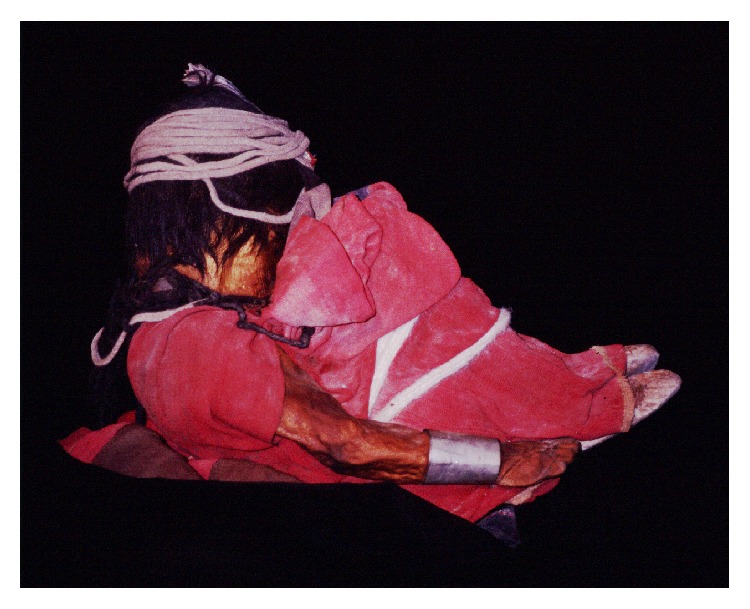
Inca mummy of the Llullaillaco boy (© Constanza Ceruti).

**Figure 14:**
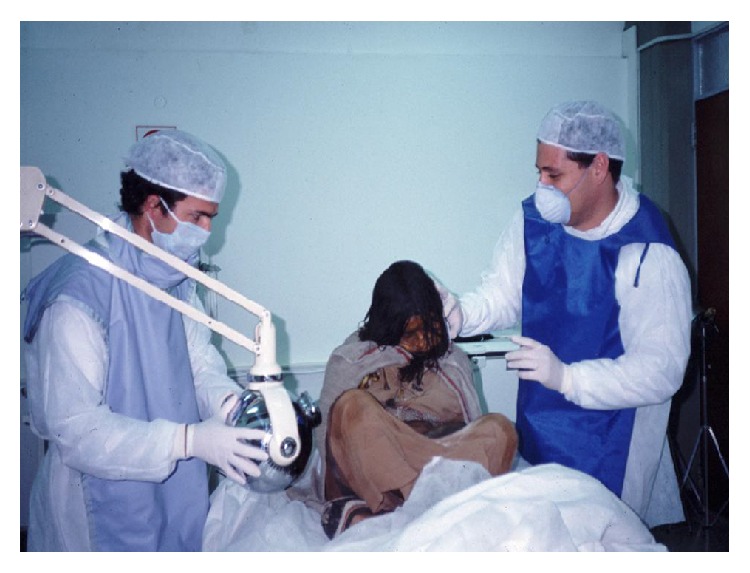
Interdisciplinary studies on the Llullaillaco maiden at the Catholic University of Salta (© Constanza Ceruti).

**Figure 15:**
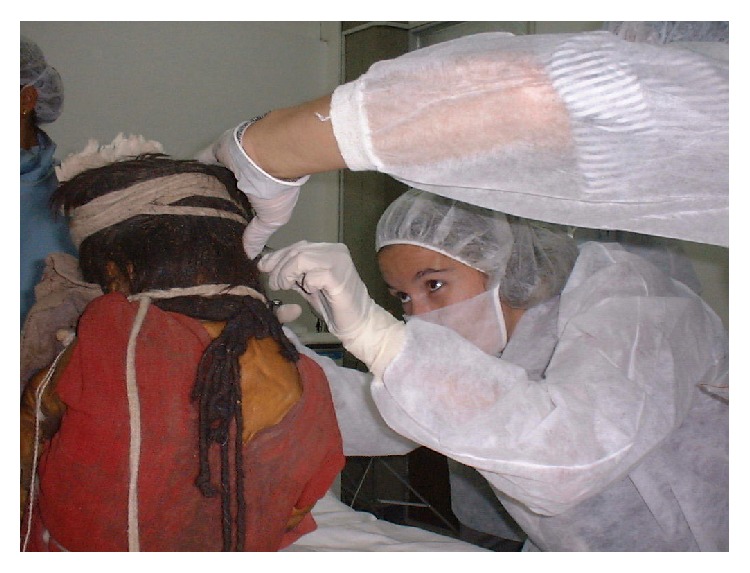
Interdisciplinary studies on Llullaillaco mummies (© Constanza Ceruti).

**Figure 16:**
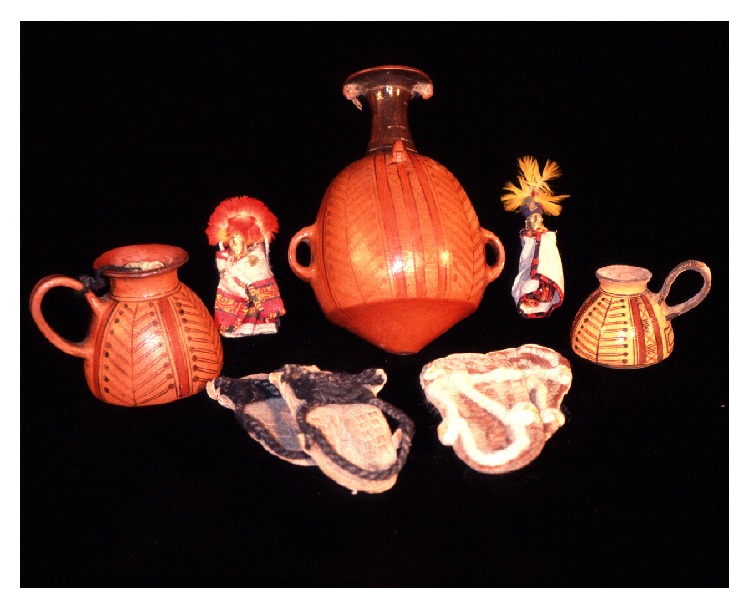
Inca offerings from mount Llullaillaco (© Constanza Ceruti).

The Inca Empire institutionalized a system of selection, seclusion, and redistribution of “chosen women” or* acllas*, who were taken from their homes prior to the onset of puberty and kept in special houses or* acllahuasi*. Here they were kept under the close surveillance of consecrated women known as* mamacona* who would teach the young girls to weave and to prepare chicha ([62]: 333). At the age of 14, the young women were taken out of the* acllahuasi* and some would be selected to be given as secondary wives to nobles while others would be consecrated to serve as priestesses or Wives of the Sun. However, some of the girls were chosen for the* capacocha* ([63]: 241). The young female from Mt. Llullaillaco (Figure 11), the famous “Ice Maiden” found on Mt. Ampato in Peru [64, 65] and the girl from the summit of Peru's Mt Sara Sara [66] appear to have been capacocha sacrificial victims because they all were all females of approximately 14 to 15 years of age and they were found with various ceremonial goods that are the same items that ethnohistorical sources report as being* capacocha* ritual offerings ([27]: 77; [37]: 473; [62]: 319; [24]: 96). Reinhard has noted that girls found on mountaintop burials tend to be older than boys because they would have been kept as virgins until being sacrificed ([32]: 112), whereas there was no institution equivalent to the* acllahuasi* to ensure a virginal condition on the boys.

All different types of Inca human sacrifices described in the historical sources, including* capacocha* and necropompa, have been identified archaeologically in diverse funerary contexts excavated in the Andes of Peru, Chile, and Argentina. The bioarchaeological evidences discussed in this paper can all be interpreted as the outcome of Inca ceremonies of* capacocha*. The only exception could be the mummy found on Mt. El Toro, since an adult male cannot easily be considered a classic* capacocha* offering. This would also account for absence of statues or other sumptuous offerings in his burial.

With regards to their social ranking, the chroniclers state that the children chosen for sacrifice were expected to be sons and daughters of nobles and local rulers ([27]: 78). Some of the victims were intentionally sent by their high status parents, in order to reinforce their political ties to the emperor and to be reaffirmed in their privileged positions of power ([37]: 472). From the bioanthropological perspective, the relatively good state of nutrition that the Llullaillaco children seemed to have enjoyed in life, as reflected in the thick layer of fatty tissue appearing in CT-scans as well as the absence of Harris lines in the X-rays, [12, 13], serve as indicators of the high social rank of these victims. From an archaeological perspective, the fact that the bodies were recovered in association with sumptuous items such as* cumbi* tunics that served specific diplomatic functions (i.e., only the emperor could give such a gift to a local ruler as a token of his good will) indicate the high social status of these sacrificial victims [19].

### 4.1. Sacrificial Procedures

During* capacocha* ceremonies, children could alternatively be sacrificed by strangulation ([67]: 150), by a blow to the head ([28]: 235; [61]: 233; [25]: 25), by suffocation ([62]: 263; [25]: 26), or by being buried alive ([27]: 46; [28]: 235).

Strangulation seems to have brought about the death of the two female mummies on Cerro Esmeralda [52], as well as for the man from Mt. El Toro [35]. Radiological studies have indicated that cranial trauma was the cause of death in the case of the Mt. Ampato maiden [65] as well as in the case of the female mummy found on Mt. Sara Sara [66]. This may also have been the sacrificial technique used to kill the boy on Mt. Aconcagua ([40]: 8). However, CT-scans and X-rays taken of the skulls of the three individuals from Llullaillaco indicated no evidence of trauma to the head [12, 13].

The practice of burying children alive or of suffocating them prior to burial is both compatible with the physical evidence of the Llullaillaco children [12] and the El Plomo boy [50]. Apparently, the reason for selecting this particular sacrificial procedure was rooted in the belief that only “complete” offerings were acceptable to a major deity. A victim who had shed blood would have been considered to be an “incomplete” offering in the eyes of the Incas ([62]: 263-264).

The case of the Llullaillaco boy suggests that death came to him as the result of exhaustion or high altitude sickness (or a combination of both), before he actually reached the summit. The relaxed position of his arms differs from the usual that mountain top sacrificial victims typically show (with their arms flexed over the body like in perimortem attempt to preserve body heat). The fetal position of the body and the tight wrapping of cords around his legs could also have been induced to facilitate the transportation of the corpse to the summit [17]. Additionally, the swap analysis of the lips of the boy has demonstrated the presence of blood in the saliva, which can be interpreted as a sign of pulmonary edema [10].

The sacrifice of children by means of burying them alive was still in use during the first decades of the Hispanic invasion. Chronicler Ramos Gavilan reports that in 1598, a ten-year-old Andean girl was found by a European miner still alive three days after she had been ritually walled inside a funerary tower (*chullpa*) by the local chiefs of the village of Sicasica, near Caracollo, in the Bolivian highlands ([25]: 66). Antonio de Herrera Tordesillas's account tells of a young Andean boy who sought refuge among the Spaniards in the Xauxa Valley (in the Peruvian Sierra), after he narrowly escaped from being ritually buried alive in occasion of the death of a local chief ([30]: 67).

### 4.2. Mountaintop* Capacochas* as Commemorative, Expiatory, Propitiatory, and Dedicatory Inca Sacrifices

*Capacocha* ceremonies that took place on volcanoes located in remotely located areas of the empire, such as the arid Atacama highlands in the case of Llullaillaco, were most likely related to the commemoration of events in the life of an Inca emperor, rather than motivated by the need to ritually respond to locally occurring natural disasters. However, expiatory or propitiatory rituals conducted with the goal of affecting local conditions could have played a part in the sacrifices performed on mountains near well populated areas, such as in the case of Mt. Chañi (near Quebrada de Humahuaca) or on Mt. Chuscha (near the Calchaquí Valley, in northern Argentina).

It is clear that both the archaeological and historical evidence support the hypothesis that human sacrifices seeking atonement were conducted on sacred summits in response to natural calamities (such as volcanic eruptions) in the hopes that such actions would put an end to the particular phenomena that was causing strife. This seems to have been the case with the offerings and human sacrifices performed inside the crater of the active Misti Volcano [19, 68].

The propitiation of fertility is suggested by the presence of* Spondylus* shell in the offerings recovered in the burials of the sacrificial victims on mounts Llullaillaco, Quehuar, Esmeralda, Chuscha, and Aconcagua. From pre-Inca times to this very day,* Spondylus* has been a ritual material of high symbolic importance in the Andes since it is believed to be related to both seawater and rain [22, 41]. Concerns about the fertility of camelids appear to be physically expressed in the assemblages of figurines on Llullaillaco and Misti, which seem to represent a caravan of llamas [17, 19].

Dedicatory rituals involving human sacrifice appear to have been performed by the Inca on mountaintop shrines because the burial of victims at these locations would have consecrated the summit ([37]: 473) and such actions would place the massif's “natural sacredness” under the domain of the imperial cult [17]. Human sacrifices on mountains conducted at the southern border of the Inca territory (like at Aconcagua and at El Plomo) could also have been related to the consecration of boundaries during the expansion of the empire [40, 69].

### 4.3. Ideological Manipulation and Political Implications of Human Sacrifice on Mountains

Sacrificial techniques employed in Inca* capacocha* ceremonies seem to have been selected in order to avoid an overt display of cruelty. This was very different from Mesoamerican heart excision and the flaying, for these extremely bloody rituals were conducted in front of a large and presumably terrified audience. Contrastingly, Inca sacrificial victims were transported to largely uninhabited and inaccessible localities and were dispatched with a minimum amount of suffering (with the spilling of blood being avoided).

We can even infer that active sacrificial procedures (such as a blow to the head or strangulation) would only have been used by the priests as a last resort, as when death caused by exposure to the cold took too long to occur or if resistance was offered by the children. The sacrificial role of the priests was apparently reduced to a minimum, making the high altitude mountain environment (with its low temperatures and extreme atmospheric conditions) a key participant in the process of bringing about the deaths of the victims. In other words, the ritual was designed to allow the mountain deity to actually take the life of the victim with the Inca religious attendants serving as subordinate facilitators in the sacrificial ritual.

As stated above, the justification for local children to be offered on mountaintop shrines during Inca* capacocha* ceremonies would have been provided by commemorative, propitiatory, or expiatory circumstances such as important events in the life of the Inca emperor, the universal Andean concern to appease the mountain deities who controlled both weather and fertility, or the need to stop locally occurring natural disasters.

The Inca sacrifice of Andean children would have been ideologically presented as having an important “mission” in which these “chosen ones” would continue living among the celestial and mountain deities as intercessors on behalf of the Inca emperor and for their own people. Their parents, their kinsmen, and local communities would have been encouraged by the Inca to believe that the payment of this type of “tribute” (consisting of their own children) was not only a religious obligation but more importantly, it was a great social “honor” as well [17].

The political implications of sacrificial ceremonies would have been utilized by local chiefs as mechanisms for creating and maintaining alliances with imperial Cuzco. Chronicler Hernandez Príncipe specifically mentions that a local leader from the Peruvian sierra (seeking to increase his political standing), volunteered his ten year old daughter for the state sponsored ritual of* capacocha* and was granted special privileges by a grateful Inca emperor as a result of his “generosity” [37].

The spectacle of these very public processions comprised of sacrificial victims (traveling with prestigious ceremonial items) radiating out from Cuzco towards the provinces would have contributed to the integration of the Inca territory by means of an intensification of the economic, political, and religious links between the center and the periphery of the empire [70–72].

## 5. Conclusions

In this presentation, we have focused on the frozen bodies from Inca high altitude shrines as objects for bioarchaeological and ethnohistorical research, providing an overview on the procedures and justifications of human sacrifice among the Incas. According to the historical sources written during the Hispanic conquest, the Inca human sacrifices were performed in response to natural catastrophes, the death of the Inca emperor, or to propitiate the mountain spirits that grant fertility. The selected children and the young* acllas* or “chosen women” were taken in processions to the highest summits of the Andes to be sacrificed. They were believed to become messengers into the world of the mountain deities and the spirits of the ancestors.

The sacrifice of young individuals on Andean mountaintop shrines at Llullaillaco, Quehuar, Aconcagua, Ampato, El Plomo, Chañi, Misti, and Chuscha was designed to celebrate special events, to ensure the well-being of the emperor, to mark a ruler's passing into the afterlife, to promote agricultural prosperity, to consecrate a specific construction project or location, to appease the deities, or to atone for sins. The Inca attributed commemorative, expiatory, propitiatory and or dedicatory motives to these sacrifices and this made the acceptance of the* capacocha* ritual by local Andean communities more feasible.

The ritual violence (inherent to Inca human sacrifice) was cloaked in a bloodless ritual conducted on remote mountaintops and was based on belief in the efficacy of sacrificed children as supernatural mediators to the mountain deities. Additionally, the “propaganda” value of convincing local elites that it was indeed an “honor” to have one's offspring sacrificed in the* capacocha* ceremony would have constituted a very efficient ideological foundation for ensuring cooperation from local rulers during the phases of expansion and consolidation of the Inca Empire.
